# The Expression of microRNA and microRNA Clusters in the Aging Heart

**DOI:** 10.1371/journal.pone.0034688

**Published:** 2012-04-18

**Authors:** Xiaomin Zhang, Gohar Azhar, Jeanne Y. Wei

**Affiliations:** Donald W. Reynolds Department of Geriatrics, The University of Arkansas for Medical Sciences and Geriatric Research, Education and Clinical Center, Central Arkansas Veterans Healthcare System, Little Rock, Arkansas, United States of America; University of Texas Health Science Center at San Antonio, United States of America

## Abstract

**Background:**

The microRNAs have been implicated in the process of cardiac development, cardiac hypertrophy, and heart failure. However, the impact of adult aging on cardiac expression of miRNA clusters, as well as both miRNA guide (miR) and passenger (miR*) strands has not been well established.

**Methods/Results:**

We explored the expression profile of both miR and miR* in the hearts of young adult versus old mice. We found that 65 miRNAs were differentially expressed in the old versus young adult hearts; approximately half of them were clustered miRNAs that were distributed in 11 miRNA clusters. Each miRNA cluster contained from 2 to as many as 71 miRNA genes. The majority of the clusters displayed similar expression, with most cluster members within a cluster being either increased or decreased together, suggesting that most clusters are likely to be regulated by a common signaling mechanism and that the combined expression of multiple miRNA genes in a cluster could pose an impact on a broad range of targets during aging. We also found age-related changes in the expression of miR*s. The expression of both miR and miR* correlated with that of pri-miRNA transcript over the time course from development and maturation through adult aging. Age-related changes in the expression of Ago1 and Ago2 proteins in the heart were also observed. Transfection assay revealed that both Ago1 and Ago2 synergistically induced miR-21 and miR-21* when the mir-21 plasmid was co-transfected with either.

**Conclusion:**

The data revealed age-related changes in the expression of pri-miRNA transcript, Argonaut proteins and both miR and miR* strands. The major changes occurred later in life, from middle to old age. It is likely that the expression of miR and miR* is regulated by both pri-miRNA transcription as well as Ago1 and Ago2 proteins during adult aging.

## Introduction

Adult aging is a complex biological process associated with a progressive decline in the physiological and biochemical performance of individual tissues and organs, leading to increased susceptibility to age-related disease and functional senescence [1]–[4]. The functional changes observed in the older heart are usually associated with morphological changes that occur during the normal aging process, which is characterized by a loss of myocytes with subsequent hypertrophy of the remaining viable myocytes, and proliferation of cardiac fibroblasts. As myocytes are lost and fibroblasts continue to proliferate and produce collagen, the physical properties of the aging heart become altered [1], [2]. The functional and morphological changes in the older heart are accompanied by changes in cardiac gene expression, which include α-MHC, β-MHC, ANF, SERCA2 etc. [5]–[10].

Recent studies suggest that microRNAs (miRNAs) may play a role in the regulation of gene expression in the aging process [11]–[13]. MiRNAs are short (20 to 23-nucleotide), endogenous, single-stranded RNA molecules that regulate gene expression by hybridization to messenger RNAs (mRNAs) with the consequence of messenger RNA degradation or translational inhibition of targeted transcripts. Genes that encode for miRNA are distributed across chromosomes either individually, or in clusters, in which two or more miRNA genes are located within a short distance on the same segment of a chromosome.

The miRNA gene is usually transcribed by RNA polymerase II into a primary miRNA (pri-miRNA) transcript, which is cleaved at a hairpin-stem by the nuclear microprocessor complex containing Drosha and DGCR8 proteins, and generates hairpin-shaped pre-miRNA. The pre-miRNA is then exported from the nucleus to the cytoplasm, where it is cleaved by Dicer to form a 22 nt double-stranded miRNA duplexes [14]. Following their production, miRNA duplexes are sorted to confer association with specific members of the Argonaute (Ago) family of proteins, which function as the core of the RNA-induced silencing complex (RISC) [15]–[20]. Post-transcriptional regulation of gene expression is mediated by the RISC complex, which uses one strand of the miRNA molecule (miR, the guide strand) to target relevant messenger RNAs. Once the messenger RNA is targeted by miRNAs, the RISC is thought to inhibit protein production either through blocking translation or by reducing messenger RNA stability [21], [22]. Previously, the microRNA passenger strand (miR* or miRNA*) was thought to be degraded without significant biological impact on gene regulation. Recent studies, including deep-sequencing efforts, however, indicate that a large number of miR*s are not simply degraded, but rather, associate with Ago1 and/or Ago2 and are also capable of silencing target transcripts [15], [16], [18], [19].

To determine the impact of advancing age on microRNA expression in the heart, we examined the microRNA expression level in old versus young adult (YA) hearts. We found that the expression of 65 microRNAs, including 11 miRNAs clusters was significantly altered during adult aging. In addition, we found that major changes in the expression of miRNA occurred from the post-maturational period through adult aging. Our data suggest that transcriptional regulation of microRNA transcripts and altered expression of Argonaut proteins contribute to age-related changes in the expression of both miR and miR* strands.

## Materials and Methods

### Animal tissues

Healthy C57BL/6 mice were obtained from colonies maintained by the National Institute of Aging (NIA), the National Institutes of Health, under contractual agreement with Harlan Sprague-Dawley, Inc. (Harlan, IN). After euthanasia, the hearts were removed from mice and subjected to standard RNA isolation and histological procedures. Some heart tissue samples (4 months and 24 months) were obtained from the Aged Rodent Tissue Bank at NIA. For each time point, there were three independent biological replicates. The studies were conducted with the approval of the Institutional Animal Care and Use Committee (IACUC) at Central Arkansas Veterans Healthcare System (IACUC# 4-02-03) and in accordance with the NIH Guiding Principles for Research Involving Animals and Human Beings.

### Total RNA isolation

All RNA samples were first isolated from the mouse cardiac ventricles using UltraSpec RNA Isolation Reagent as previously described. To minimize mouse DNA contamination and enrich the small RNA fraction, the total RNA samples were purified using miRNeasy Mini kit (Qiagen) and RNase-free DNase I according to the manufacture's instruction manual [23], [24].

### MicroRNA arrays

The ventricular tissue samples used for the microRNA array analysis were obtained from healthy young adult (4-month-old) and old (24-month-old) C57BL6 mice. The RNA sample isolation and microRNA array were performed in triplets for young adult and old animals. A total of 6 RNA samples of ventricular tissue representing 3 individual young adult and 3 individual old mice were shipped on dry ice to Exiqon, Inc., which provided the service for RNA quality verification, microRNA array hybridization and comprehensive statistical analysis. The microarray data have been deposited in the NCBI Gene Expression Omnibus (GEO) database (http://www.ncbi.nlm.nih.gov/geo/) under accession no. GSE32935. Briefly, each pair of young adult and old mouse samples were labeled with Hy3 and Hy5 fluorescent dyes respectively and hybridized to a miRCURY LNA™ mouse microRNA Array (version 11.0), which held 648 mature microRNA probes, as well as perfectly matched and mismatched probes for quality control. After signal amplification, the background was subtracted and normalized using LOWESS (Locally Weighted Scatter plot Smoothing) regression algorithm. This within-slide normalization was performed to minimize differences between the colors in an intensity-dependent manner. The array output was received in Excel spreadsheets containing the normalized microRNA expression profiles in each heart sample; the expression comparison between old versus young adult heart samples and “Expression Matrix” containing normalized Hy3/Hy5 ratios (log2 transformed) from all hybridizations. The list was sorted based on the most variant expressed miRNAs comparing the two sample types. 65 miRNAs passed the filtering criteria with an average “log-Median-Ratio” >0.58, which represents at least >1.5-fold change in microRNA expression, and the microRNA expression in all three pairs (young adult versus old) was in the same direction.

### Real-time RT-PCR quantitation of pri-miRNAs and mature miRNAs

To quantitate the expression of the miRNA primary transcripts and the miRNA mature forms, Real-time RT-PCR was performed. To select a proper internal loading control for RT-PCR, we examined the expression of 5S ribosomal RNA (5S RNA) and U6 snRNA in young adult versus old hearts. We found that the expression of 5S RNA remained unchanged in young adult vs. old, while U6 expression changed significantly in young adult vs. old hearts. Therefore, 5S RNA was used as an internal loading control.

#### Detection of pri-miRNAs

The primers for the detection of pri-miRNAs were designed using PRISM Primer Express 3.0 software (Applied Biosystems), and synthesized at Integrated DNA Technologies Inc. The first-strand cDNA synthesis was carried out using random hexamer primer, and the PCR was performed using the following primers: pri-mir-17 forward 5′-gctttggctttttcctttttg-3′, pri-mir-17 reverse 5′-cctcactgcagtagatgcaca-3′; pri-mir-21 forward 5′-ccagagatgtttgctttgctt-3′, pri-mir-21 reverse 5′-tgccatgagattcaacagtca-3′; pri-mir-27a forward 5′- tttgatgccagtcacaaatca-3′, pri-mir-27a reverse 5′-agccactgtgaacacgacttt-3′; pri-mir-93 forward 5′-cacctcacctaatgaccctca-3′, pri-mir-93 reverse 5′-caagtcctagccctcatggat-3′; pri-mir-466d forward 5′- cacatgcaacacacacatatgaa-3′ and pri-mir-466d reverse 5′-ctgattctggcaagcattttc-3′.

#### Detection of mature miRNAs

To detect the mature miRNA strand of either miR or miR*, the first-strand cDNA synthesis was carried out using a universal reverse primer and the Universal RT microRNA PCR system (Exiqon). The RT-PCR reagents and the primers for miR-17, miR-21, mir-21*, miR-27, miR-93, miR-466d-3p and the 5S RNA reference primers that were used as endogenous control were purchased from Exiqon.

The PCR amplification was performed in a 7900HT Fast Sequence Detector System (Applied Biosystems) with following program: Cycle 1, 95°C for 10 minutes. Cycle 2, 40 cycles of 95°C for 15 seconds, 60°C for 60 seconds. Cycle 3, 95°C for 15 seconds, 60°C for 15 seconds, 95°C for 15 seconds. CT values were automatically obtained. Relative expression values were obtained by normalizing CT values of the miRNA genes in comparison with CT values of the endogenous control (5S RNA) using the CT method [25], [26].

### Transfection Assays

The Ago1 expression plasmid is a gift of Dr. E. K. L. Chan, and the Ago2 expression plasmid is a gift of Dr. T. Tuschl. The plasmid construct that contains the mir-21 gene was generated by PCR and cloning. Briefly, the DNA fragment containing mir-21 gene was amplified using forward primer 5′- tagaattc tgcccaggcttttatgtattg -3′ and reverse primer 5′- ta ctcgag ggcattgcttttcaagtatgg -3′, and then cloned into pCDNA3.1(+) vector. The DNA construct was confirmed by sequencing analysis.

The cardiac muscle cell line H9C2 was cultured in DMEM medium containing 10% newborn bovine serum (Invitrogen). Transient transfections were carried out with the Lipofectamine 2000 reagents (Invirogen) as previously described [24]. At 4 h after the transfection was initiated, the H9C2 cells were placed in Dulbecco's modified Eagle's medium with 10% newborn bovine serum and incubated overnight. The cells were then harvested and the total RNA was isolated using miRNeasy Mini kit (Qiagen) and RNase-free DNase I according to the manufacture's instruction manual. Individual transfection experiments were carried out in triplicate, and the results were reported as average (mean ± S.D.) from representative experiments.

### Statistical analysis

Data are given as mean values ± SD, with *n* denoting the number of experiments unless otherwise indicated. The differentially expressed microRNAs with at least a 1.5-fold change were identified using a t-test with a cut off p-value (p<0.05).

## Results

### 1. Differential expression of cardiac microRNAs in the aging heart

It has been reported that microRNA expression was altered in cardiac hypertrophy, congestive heart failure and also in senescent cells [27]–[31]. To test the hypothesis that the expression of microRNA may also change during cardiac aging, we compared microRNA profiles in the young adult (YA, 4-month-old) versus old (O, 24-month-od) mouse hearts. The Exiqon microRNA array (version 11.0) was employed for the microarray analysis.

**Figure 1:**
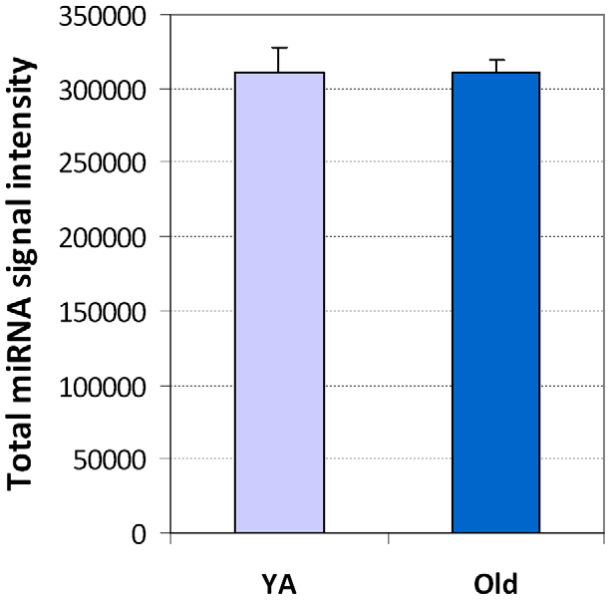
The signal intensity of miRNAs expressed in the mouse heart. There was no significant difference in the overall signal intensity of total microRNAs between young adult (YA) and old hearts.

**Figure 2:**
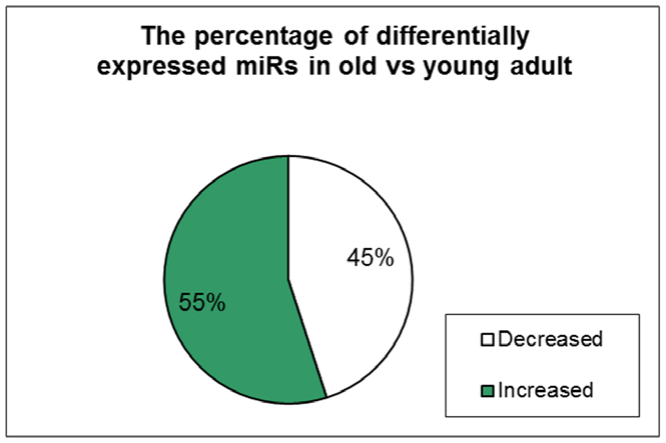
The number of miRNA that were differentially expressed in the heart. Among the 599 microRNAs that were expressed in the heart, 272 miRNAs were down-regulated, while 327 miRNAs were up-regulated in the old compared with young adult hearts.

An overview of the total microRNA expression in YA versus old hearts showed that the total microRNA signal intensity was 311248 in YA hearts versus 310447 in old hearts (Figure 1); there was no significant difference between the two age groups (NS, n = 3). However, the comparison of each individual microRNA expression between old and YA showed that 45% of the microRNAs were down-regulated in old versus YA, while 55% of the microRNAs were up-regulated in old versus YA (Figure 2, p<0,01, n = 3). These data indicate that while the expression of many of the individual microRNAs changed in advancing age, the healthy old heart was still able to keep the overall microRNA expression close to that of the YA heart.

**Figure 3:**
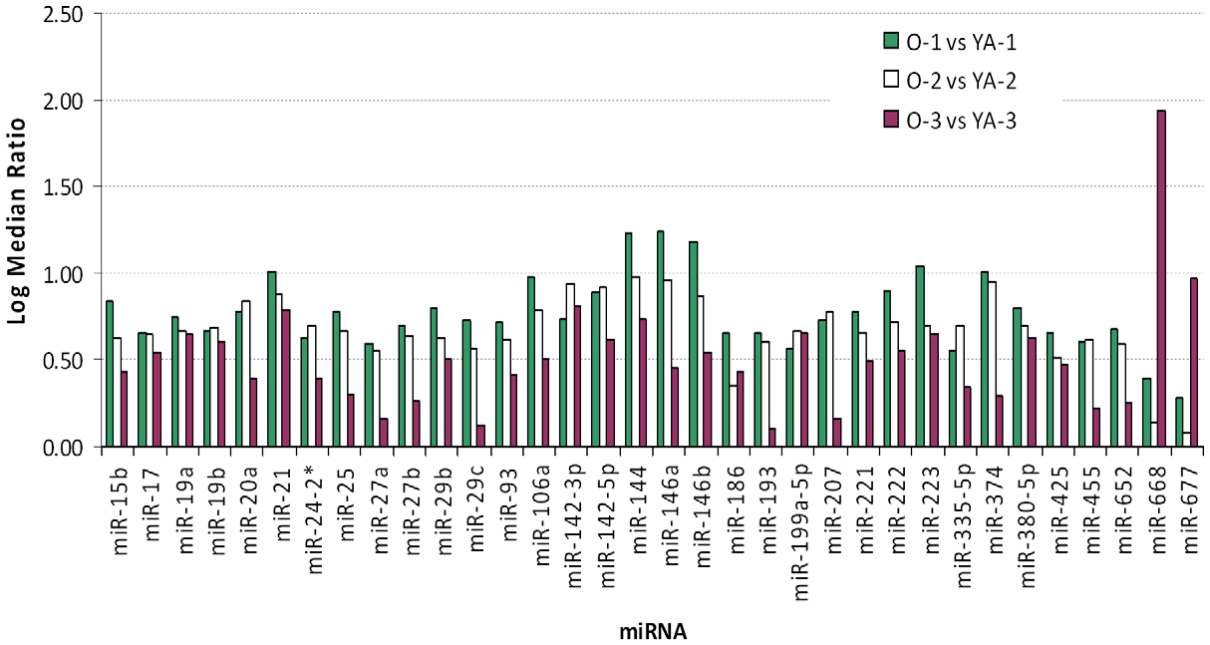
The miRNAs that were increased during adult aging. Thirty-four miRNAs were increased in old (O) vs. young adult (YA) hearts.

**Figure 4:**
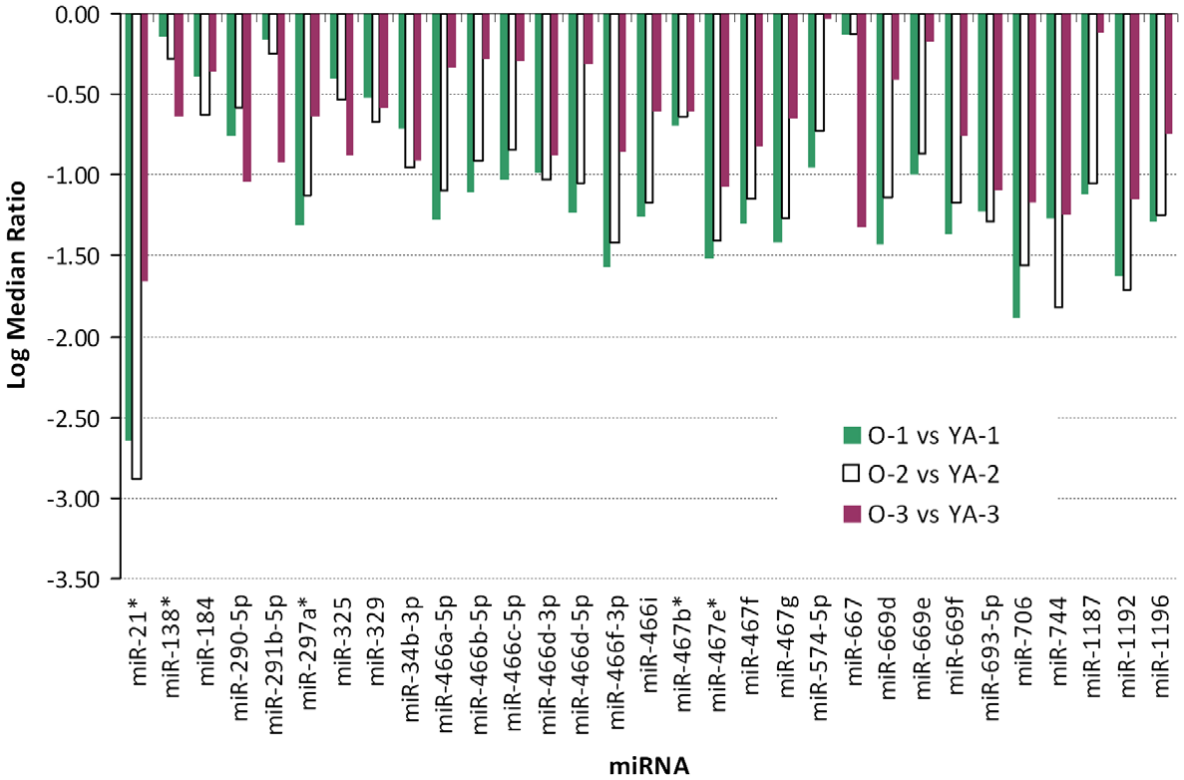
The miRNAs that were decreased during adult aging. Thirty-one miRNAs were decreased in old (O) vs. young adult (YA) hearts.

To examine the miRNAs that were significantly altered during aging, the expression of miRNAs in YA versus old hearts were compared. It was found that the expression level of 65 miRNAs changed over 1.5-fold in old compared to that of YA hearts. Among them, 34 miRNAs were up-regulated, while 31 were down-regulated (Table 1 and Figure 3, 4).

**Table 1:** The 65 miRNAs that were differentially expressed in old versus young adult hearts.

Increased	Decreased
miR-15b, miR-17, miR-19a, miR-19b,	miR-21*, miR-138*, miR-184,
miR-20a, miR-21, miR-24-2*, miR-25,	miR-290-5p, miR-291b-5p, miR-297a*,
miR-27a, miR-27b, miR-29b, miR-29c,	miR-325, miR-329, miR-34b-3p,
miR-93, miR-106a, miR-142-3p	miR-466a-5p, miR-466b-5p,
miR-142-5p, miR-144, miR-146a,	miR-466c-5p, miR-466d-3p,
miR-146b,miR-186, miR-193,	miR-466d-5p, miR-466f-3p, miR-466i,
miR-199a-5p, miR-207, miR-221,	miR-467b*, miR-467e*, miR-467f,
miR-222, miR-223, miR-335-5p,	miR-467g, miR-574-5p, miR-667,
miR-374, miR-380-5p, miR-425,	miR-669d, miR-669e, miR-669f,
miR-455, miR-652, miR-668, miR-677	miR-693-5p, miR-706, miR-744,
	miR-1187, miR-1192, miR-1196

34 miRNAs were increased, while 31 miRNAs were decreased in old heart.

### 2. Multiple microRNA clusters are affected in the aging heart

The miRNA genes are distributed across chromosomes either individually, or in clusters. A miRNA cluster is a group of miRNA genes located within a short distance on a chromosome. Based on miRBase database (http://www.mirbase.org) definition, clustered miRNAs are a group of miRNA genes located within 10 Kb of distance on the same chromosome. To test the hypothesis that adult aging may affect both individually distributed miRNAs and the microRNAs in clusters, we examine the genomic location of the 65 miRNAs that were differentially expressed in the aged heart. We found that approximately one half of the differentially expressed miRNAs was individually distributed and the other half was in miRNA clusters.

**Figure 5:**
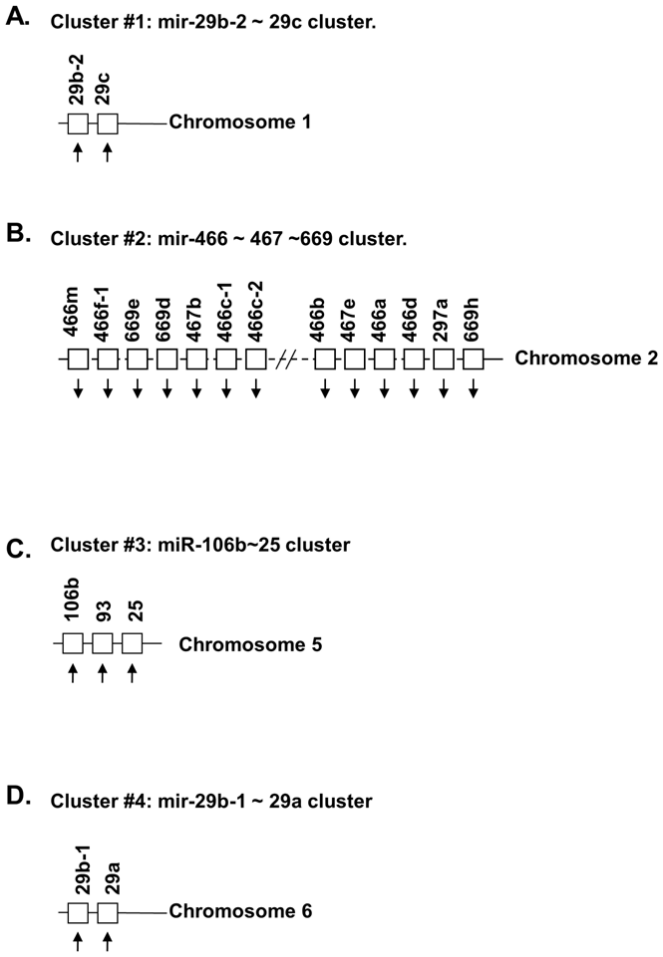
The miRNA gene clusters and their genomic structures of cluster #1∼cluster #4, which are part of 11 miRNA gene clusters shown in figure 5, 6 and 7. The number of miRNA genes in each cluster ranged from 2 to 71. In 10 out of 11 clusters, the expression of most miRNA genes in that cluster was in the same direction (the arrow **↑** for increased expression, and the arrow **↓** for decreased expression). Please note that part of the miRNAs with changes in expression greater than 1.5 fold in old (O) vs. young adult (YA) hearts were listed in table 2.

**Figure 6:**
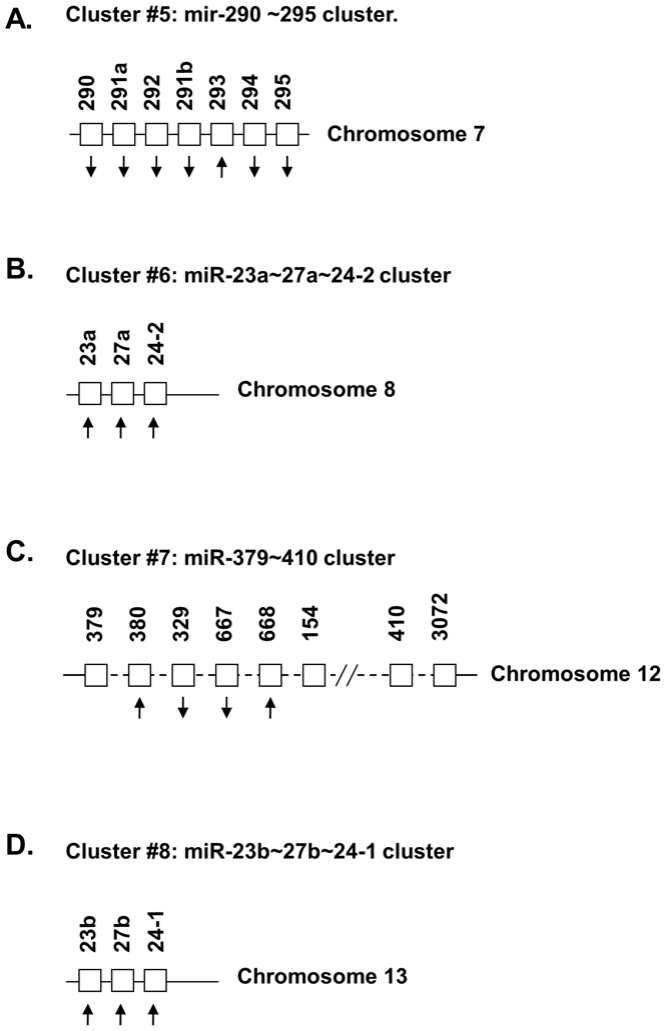
The miRNA gene clusters and their genomic structures of cluster #5∼cluster #8, which are part of 11 miRNA gene clusters shown in figure 5, 6 and 7. The number of miRNA genes in each cluster ranged from 2 to 71. In 10 out of 11 clusters, the expression of most miRNA genes in that cluster was in the same direction (the arrow **↑** for increased expression, and the arrow **↓** for decreased expression). Only in one cluster, cluster #7 (Figure 6C), were most of the miRNA genes not expressed in the same direction. Please note that part of the miRNAs with changes in expression greater than 1.5 fold in old (O) vs. young adult (YA) hearts were listed in table 2.

**Figure 7:**
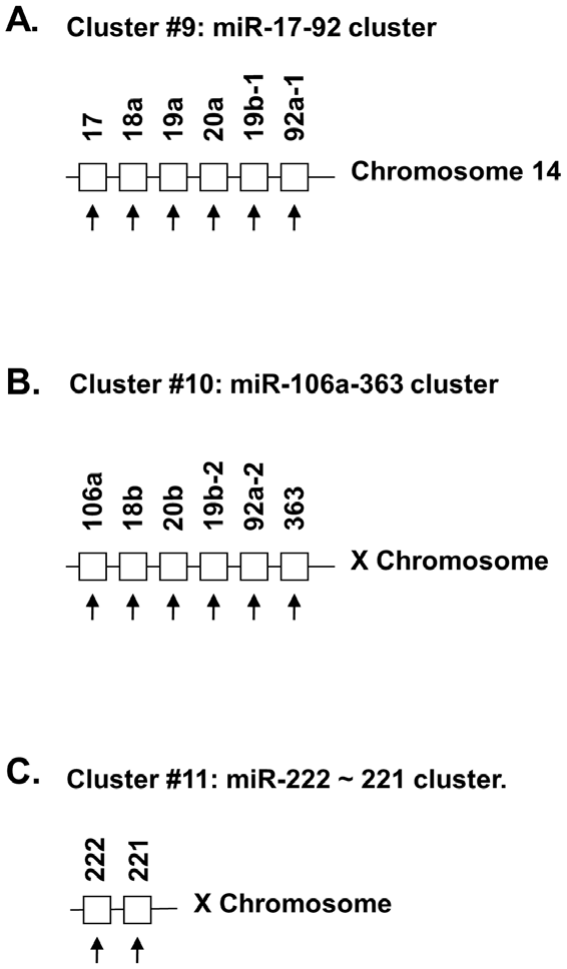
The miRNA gene clusters and their genomic structures of cluster #7∼cluster #11, which are part of 11 miRNA gene clusters shown in figure 5, 6 and 7. The number of miRNA genes in each cluster ranged from 2 to 71. In 10 out of 11 clusters, the expression of most miRNA genes in that cluster was in the same direction (the arrow **↑** for increased expression, and the arrow **↓** for decreased expression). Please note that part of the miRNAs with changes in expression greater than 1.5 fold in old (O) vs. young adult (YA) hearts were listed in table 2.

**Figure 8:**
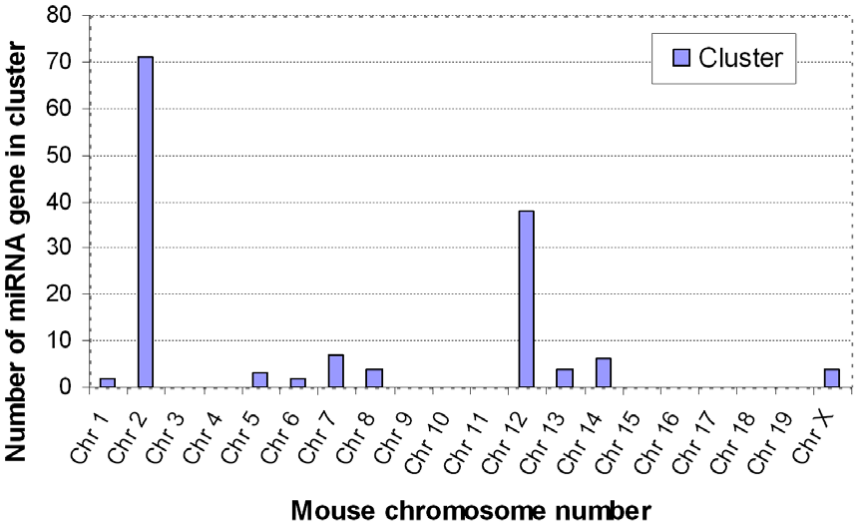
The 11 miRNA clusters that were affected during adult aging are located on 10 chromosomes. Mouse×Chromosome contains 2 clusters (cluster #10 “mir-106a-92a-2” with 6 miRNA genes, and #11 “mir-222∼221” with 2 miRNA genes).

The 32 miRNAs shown in Table 2 had 1.5-fold change in expression in old compared to that of YA hearts. The miRNA genes that corresponded to the 32 miRNA mature forms were in 11 clusters with their other cluster member(s) (Figure 5, 6, 7 and 8). Each clusters had at least 2 genes, with mir-466∼467∼669 cluster (cluster # 2) having 71 genes and mir-379∼410 cluster (cluster #7) having 38 genes. Since genes in a miRNA cluster may share a common gene promoter and may be regulated as a whole transcriptional unit, it could be of interest to determine whether any entire cluster changed in the same direction, either up or down. After examination, it was found that the majority of the miRNAs in 10 of the 11 clusters (all clusters except cluster #7, Figure 6C) tended to change in the same direction as the other members in that cluster (Figure 5, 6 and 7), although not all of them had a 1.5-fold change (p<0.01) in expression, and not every change was significant (p>0.05).

**Table 2:** The 32 miRNAs and their corresponding miRNA gene clusters.

Cluster #	miRNA cluster	miRNAs differentially expressed in O vs YA hearts
#1	mir-29b-2∼29c	miR-29c
#2	mir-466-467-669	miR-466a-5p, miR-466b-5p, miR-466c-5p
		miR-297a*, miR-466d-3p, miR-466d-5p,
		miR-466f-3p, miR-466i, miR-467b*,
		miR-467e*, miR-467f, miR-467f,
		miR-467g,miR-669d, miR-669e, miR-669f
#3	mir-106b-93-25	miR-106a
#4	mir-29b-1∼29a	miR-29b
#5	mir-290∼293∼295	miR-290-5p, miR-291b-5p
#6	miR-23a∼27a∼24-2	miR-27a
#7	mir-379∼411∼758	miR-380-5p
#8	mir-23b-27b-24-1	miR-27b
#9	mir-17-19a-92a-1	miR-17, miR-19a, miR-19b, miR-20a
#10	mir-106a-92a-2	miR-25, miR-93,
#11	mir-222∼221	miR-221, miR-222
**Total: 11 miRNA clusters**		**32 miRNAs**

Approximately half of the miRNAs (32 out of 65 miRNAs) that were differentially expressed in the old heart belong to 11 miRNA gene clusters. These 32 miRNAs had greater than 1.5-fold change in old (O) versus young adult (YA) hearts. Other miRNAs in the clusters with less than 1.5-fold change in expression are not shown in this table.

### 3. Age-related changes in the expression of miRNA guide strands and their corresponding primary transcripts

**Figure 9:**
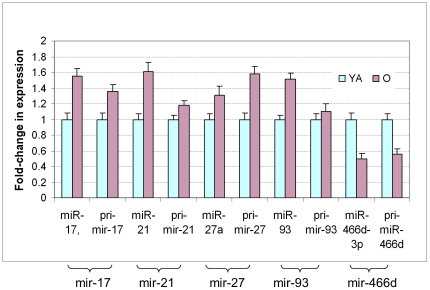
The expression of miRNAs correlated with that of their corresponding pri-miRNAs. The mature form of miRNAs: miR-17, miR-21, miR-27 and miR-93 were up-regulated, as were their underlying pri-miRNAs in the old (O) vs. young adult (YA) hearts. The miR-466d-3p was down-regulated, as was also its pri-mir-466d.

The transcription of pri-miRNA is the first step in miRNA biogenesis [32]. Pri-miRNA is first cleaved by the microprocessor complex into pre-miRNA, which is then cleaved by the RNase III enzyme Dicer and become mature microRNAs as a guide strand (miR) and a passenger strand (miR*). During typical miRNA biogenesis, the miR strand is preferentially selected for entry into a RISC complex, whereas the miR* strand could be degraded [33]–[35]. To test the hypothesis that transcriptional regulation plays an important role in the regulation of the mature microRNA form, we examined pri-miRNA and miR with real-time PCR. As shown in Figure 9, the miRNA guide strand miR-17, miR-21, miR-27a and miR-93 had an age-related increase accordingly with their corresponding primary transcripts pri-mir-17, pri-mir-21, pri-mir-27 and pri-mir-93. Similarly, both mir-466d primary transcript and miR-466d-3p showed an age-related decrease in expression.

**Figure 10:**
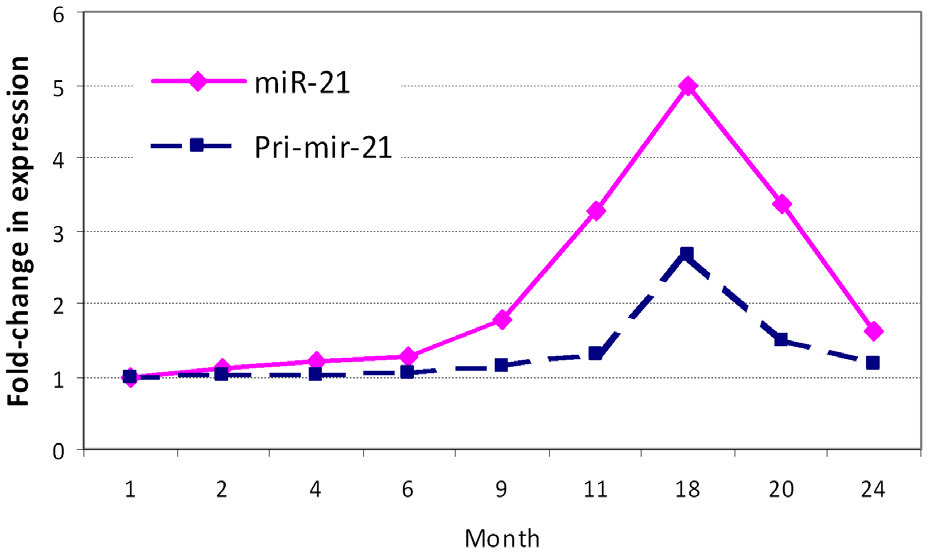
The miR-21 expression correlated with pri-mir-21 levels from 1 month to 24 months of age.

To further examine whether the expression of both pri-miRNA transcript and the miR strand change during maturation and aging, we compared the pri-mir-21 and the miR-21 levels in the mouse heart from 1 month to 24 months of age. As displayed in Figure 10, both miR-21 and pri-mir-21 levels showed no significant change from 1 month to 6 months of age, but started to increase after 6 months of age. Both miRNA forms reached their highest levels at the age of 18 months, then decreased afterwards; both curves showed a reversed “V” shape during adult aging.

### 4. Age-related changes in the expression of miRNA passenger strand (miR*) in the mouse heart

In the cytoplasm, the pre-miRNA becomes cleaved by Dicer, which results in the formation of a 22 nt double-stranded miRNA duplex, with one being the guide strand (miR) and the other, the passenger strand (miR*). Recent deep-sequencing efforts indicate that a large number of miR*s are not degraded, but rather associate with Ago1 or Ago2 and remain functional [15], [17]–[20].

**Figure 11:**
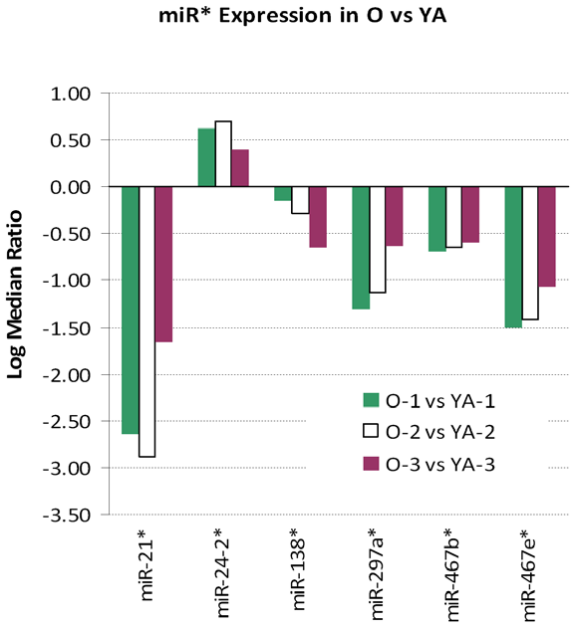
Altered expression of miR* during adult aging. Six miR*s that were differentially expressed in old (O) vs young adult (YA) hearts.

An age-related change in the expression of miRNA passenger strands was observed in the present study. Five miR*s: miR-21*, miR-24-2*, miR-138*, miR-297a*, miR-467b* and miR-467e*, were decreased at least 1.5-fold, while miR-24-2* was increased at least 1.5-fold in old versus YA hearts (Figure 11). Interestingly, the fold of change in expression of miR-21* was the highest among all 65 miRNAs that were differentially expressed in old versus YA hearts (Figure 4 and 11).

**Figure 12:**
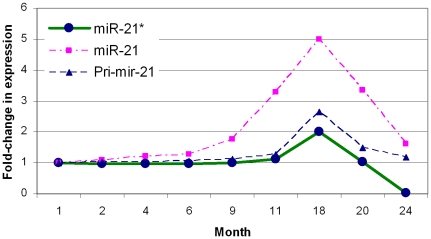
The miR-21* passenger strand expression in comparison with miR-21guide strand and pri-mir-21 transcript in mouse hearts, from 1 month to 24 months of age.

The level of miR-21* strand was examined in comparison with miR-21 and pri-mir-21 transcript from 1 month to 24 months of age. As shown in Figure 12, the miR-21* expression level did not undergo significant change from 1 month to 9 months of age, but increased thereafter, and reached the highest level at age 18 months. However, its expression level reduced to its lowest level at 24 months of age. The miR-21* expression curve during adult aging resembles a reverse “V” shape (Figure 12), which is similar to what was observed in the miR-21 and pri-mir-21 expression curves. These data suggest that the primary transcript expression level affects both miR-21 and miR-21* levels.

Bioinformatic analysis using MicroCosm Target Scan (http://www.ebi.ac.uk/enright-srv/microcosm/htdocs/targets/v5/#) revealed that mmu-miR-21* had 879 potential target hits, while Mmu-miR-21 had 894 hits, 95% of them are not overlapped. This finding suggest that the passenger strand is not always passive, but at least in some cases, has an important regulatory role in gene expression as well.

### 5. Age-related change in the expression of Ago1 and Ago2

**Figure 13:**
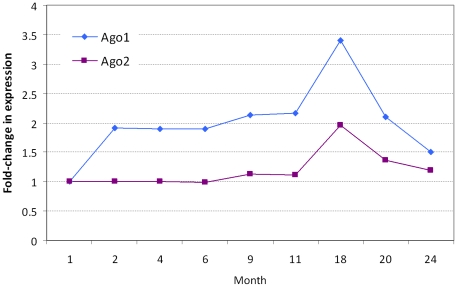
The expression of Argonaute genes. The cardiac expression of Ago1 and Ago2 in the mouse, from 1 month to 24 months of age.

Argonaute proteins have endonuclease activity directed against messenger RNA strands that are complementary to their bound miRNA fragments. Argonaut proteins are also partially responsible for selection of the guide strand and destruction of the passenger strand. To test the hypothesis that Argonaute expression may change, thereby affecting both miR and miR* expression level during adult aging, we examined the expression of Ago1 and Ago2 genes during development, maturation and adult aging by real-time RT-PCR. As shown in Figure 13, Ago1 expression level was increased from 1 to 2 months of age and remained at that level from 2 months to 11 months, then reached the highest level at age of 18 months. Afterward, its level was decreased. The Ago2 expression stayed at the same level from 1 month to 11 months of age, increased after 11 months of age and reached the highest level at 18 months of age. It then was also reduced after 18 months. Both the Ago1 and Ago2 expression levels resembled a reversed “V” shape during adult aging from 11 months to 20 months of age.

**Figure 14:**
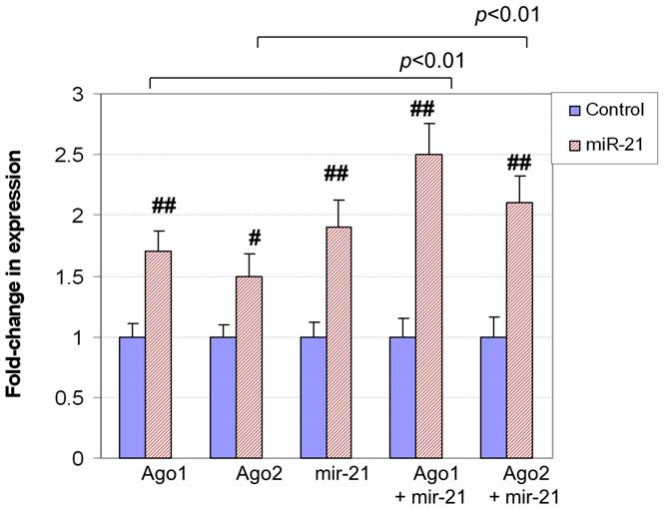
The effect of Ago1 and Ago2 on the expression of miR-21. In the transfection assays, the cells were transfected with empty vector (Control), or Ago1, Ago2, mir-21, Ago1 and mir-21 as well as Ago2 and mir-21. The miR-21 guide strand was upregulated by Ago1, Ago2, mir-21 gene, Ago1 and mir-21 as well as Ago2 and mir-21 gene. The data indicated that Ago1 and Ago2 synergistically upregulated miR-21 guide strand when mir-21 gene was increased. # refers to p<0.05, n = 3. ## refers to p<0.01, n = 3.

**Figure 15:**
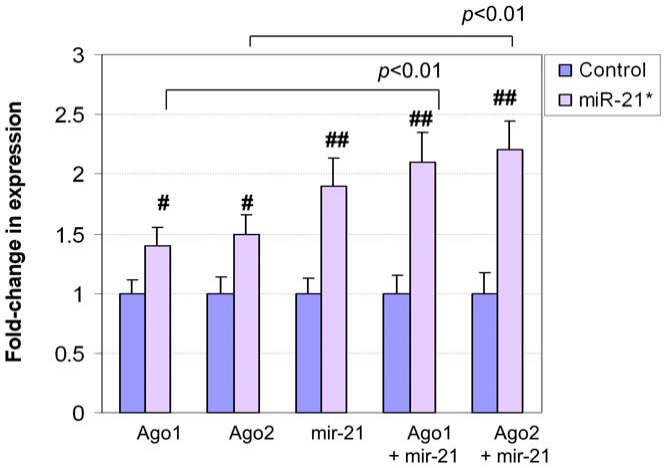
The effect of Ago1 and Ago2 on the expression of miR-21*. In the transfection assays, the cells were transfected with empty vector (Control), or Ago1, Ago2, mir-21, Ago1 and mir-21 as well as Ago2 and mir-21. The miR-21* passenger strand was upregulated by Ago1, Ago2, mir-21 gene, Ago1 and mir-21 as well as Ago2 and mir-21 gene. The data indicated that Ago1 and Ago2 synergistically upregulated miR-21* passenger strand when mir-21 gene was increased. # refers to p<0.05, n = 3. ## refers to p<0.01, n = 3.

To test the effect of Ago1 and Ago2 on the expression of miR-21 and miR-21*, the Ago1 or Ago2 expression plasmid vector was transfected into cells derived from a cardiac muscle cell line, H9C2. As shown in Figure 14 and 15, the overexpression of Ago1 or Ago2 induced the expression of miR-21 or miR-21*, respectively. In addition, both Ago1 and Ago2 synergistically induced miR-21 and miR-21* when the mir-21 plasmid vector was co-transfected with either.

## Discussion

The present study has several major findings. We found that adult aging significantly impacted the expression of 65 miRNAs in the heart; among them 55% miRNAs were increased, while 45% miRNAs were decreased in the old versus young adult hearts. Interestingly, over half of these differentially expressed miRNAs belong to 11 miRNA clusters, suggesting that these clusters participate in the complex regulation of cardiac gene expression during adult life. We also observed age-related changes in the expression of pri-miRNA transcript and Ago genes, which likely contribute to age-related changes in both miR and miR* expression in the heart. These data enhance our understanding of the complex mechanism(s) underlying the altered expression of miRNA during adult aging.

In the present study, half of the miRNAs that are significantly impacted during adult aging belong to 11 miRNA clusters, suggesting the importance of understanding the structure and function of these miRNA clusters. A miRNA cluster is a group of miRNA genes located within a short distance on a chromosome. Many of the miRNA clusters may have one core promoter region and transcriptional start site shared by all miRNA genes within that cluster and were ultimately expressed within a single RNA transcript [36]. The length of the transcript typically varies from a couple of kb to over 10 kb. For instance, the polycistronic transcript of mir-1-1 and mir-133a2 cluster is 10 kb; the mir-1-2 and mir-133a1 cluster is 6 kb [24]. It has been reported that each miRNA cluster usually has 2–3 miRNA genes, with the majority of clusters having less than 10 miRNA genes [37]. However, some clusters could have more than 10 miRNA genes. For example, Hertel reported that mir-134 cluster has more than 50 miRNA genes [37]. In the present study, we found that the miR-379∼329∼667∼410 cluster has 38 mir genes, while the miR-466∼467∼669 cluster, being one of the largest miRNA clusters in mouse genome, has 71 miRNA genes. Although a number of miRNA clusters have been reported to be associated with pathological and physiological conditions, the overall expression pattern of miRNA clusters during adult aging has not been extensively studied. Since many miRNA genes within a cluster share a common promoter or utilize a common regulatory machinery, examination of their expression pattern and regulatory mechanism could enhance our understanding of the role of miRNAs in the process of adult aging.

The effects of these altered microRNAs and miRNA clusters on target gene expression during adult aging and senescence warrant further investigation. Exogenous miRNAs can be introduced into live cells or animals; likewise, endogenous miRNAs can also be knocked-down or deleted in cells or animals with various approaches. However, the effects of the various approaches might not be the same [38]. It is believed that the length of the 3′ untranslated regions (3′ UTRs) of the target messenger RNAs, the cellular proliferative status, and the abundance of target messenger RNA may affect miRNA regulation [39]–[42]. For instance, functional miRNA target sites tend to reside in 3′ UTRs, therefore the length of the 3′ UTRs often correlates with density of the miRNA target sites [41], [43]. Highly proliferating cells usually express more messenger RNAs with shorter 3′ UTRs, which might escape miRNA targeting, whereas low proliferating cells, including senescent cells, may express miRNAs with longer 3′ UTRs [39]–[41]. In addition, transfected (exogenous) miRNAs may compete with endogenous miRNAs for the same protein complex, including RISC protein complex that is needed for miRNA regulation. Therefore, genes that are only targeted by endogenous miRNAs but not by the exogenous miRNA may end up relatively increased [44]. In addition, the target messenger RNA abundance could potentially dilute microRNA and siRNA activity, thereby reducing the effectiveness of miRNA regulation [45]. Nevertheless, miRNA transfection assays have been shown to target various messenger RNAs and intracellular pathways. For example, miR-494 down-regulates IGF2BP1 and induces cellular senescence in A549 cell line [46]. In neural stem cells, miR-106b∼25 target the insulin/insulin-like growth factor-1 (IGF) signaling pathways and promote neuron growth [47]. These factors (various approaches of knock-down vs. deletion, cellular proliferative status, length of 3′ UTR of target, and abundance of messenger RNA target) all contribute to the reported and potentially other differences that may result from “aging” *in vitro* or *in vivo,* in the experimental models being used.

The present study suggests that most miRNA clusters are likely to be regulated at the cluster level by certain common signaling pathway(s). The combined effects of changes in expression of an entire miRNA cluster warrant further investigation. In addition, whether the altered miRNA expression, alone or in clusters, might be causal or contributory to myocardial aging remains to be determined.

One of the 11 miRNA clusters uncovered in the present study is the cluster miR-17–92. The miR-17–92 cluster is comprised of 6 miRNA genes: miR-17, miR-18a, miR-19a, miR-20a, miR-19b-1 and miR-92–1, all of which are located on chromosome 14 in the mouse. The human ortholog of miR-17-92 is indeed expressed within a single expressed sequence tag (EST), chr13orf25. In addition, the sequence upstream of the putative transcriptional start site is highly conserved across vertebrate genomes and contains an extensive CpG island, indicative of a core promoter region [36].

The miR-17-92 cluster has been implicated not only in cell proliferation and tumor growth, but also in cellular senescence. Increased expression of miR-17-92 cluster is usually associated with oncogenesis, cell growth and proliferation, while decreased expression of miR-19 and other members of the miR-17-92 cluster are generally associated with cellular senescence [48]–[50]. However, in the older heart, decreased expression of miR-17-92 cluster had been observed only in failure-prone (C57Bl6×129Sv) mouse and human failing heart, but not in healthy aging heart [51]. In addition, the change in expression of miR-17-92 cluster is only observed in cardiac myocytes, but not in fibroblasts [51]. In the present study, the miR-17-92 cluster is up-regulated in the old heart of the C57BL6 mouse that is free of disease. This increased miR-17-92 cluster expression in the present study is likely correlated with the age-related hypertrophy of the cardiac myocytes in the old heart. It would be of interest to perform transfection assays to determine if up-regulation of this cluster might reproduce changes that would be similar to the observed age-related differences. However, it is possible that a broad range of genes would be affected if all 6 miRNA genes were to be simultaneously overexpressed by transfection. Future studies of this cluster will be helpful to delineate the effects on their target genes.

Two other clusters, miR-23a∼27a∼24-2 and mir-23b-27b-24-1, each containing three miRNA genes, were observed in the present study to be up-regulated to different degrees in the old versus young adult heart. It has been reported that the expression levels of 23a-27a-24–2 cluster members are significantly up-regulated upon treatment with isoproterenol and aldosterone, but only miR-23a participates in initiating the hypertrophic response [52]. miR-23a, miR-27b were also induced during early hypertrophic growth in response to pressure-overload [53], [54]. The miRNA genes in these clusters are important for cardiac development and maturation [55], [56]. The present study revealed that the miRNAs in these two clusters might also play a role in the cardiac aging process.

The miR-466∼467∼669 cluster is one of the largest miRNA clusters in the mouse genome, containing 71 miRNA genes. It is embedded in the intron of the Scm-like with four mbt domains 2 (Sfmbt2) gene. Sfmbt2 is a mouse and human homologue of Polycomb group (PcG) gene. Sfmbt2 is located on proximal chromosome 2, in a region known to be imprinted [57], [58]. Sfmbt2 is expressed preferentially from the paternal allele in early embryos and in later stage extra-embryonic tissues. This is partly due to the fact that a CpG island spanning the transcriptional start site is differentially methylated on the maternal allele during embryogenesis [57].

Although insight into the regulatory function of miRNAs toward their messenger RNA targets are continuing to emerge, less is currently known about the regulation of miRNA gene expression and miRNA biogenesis [14], [24]. To date, hundreds of miRNAs have been discovered in mammalian genomes. Approximately 50% of mammalian miRNAs are “intragenic miRNAs” that are located in introns or exons of protein coding genes; the other half of the miRNAs are “intergenic” miRNAs that are derived from pri-miRNAs expressed from intergenic regions where a stretch of DNA sequences located in the chromosomal region referred as junk DNA region that contain few or no protein-coding genes [59], [60]. It is generally believed that intragenic miRNAs are co-transcribed with their host genes, while most intergenic miRNAs are transcribed from their own RNA polymerase II (Pol II) promoter. However, recent studies indicate that as much as 26% of the intragenic miRNAs may be transcribed from their own unique promoters even though they are physically located within protein coding genes. The miRNA gene promoters have similar features to those of protein coding genes, but miRNA transcript organization is more complex [60]. In the present study, we observed that the expression of pri-miRNA levels change during adult aging, which correlates with the level of miRNA, suggesting that transcription regulation likely impacts miRNA biogenesis.

Argonaute proteins are key effectors of small RNA-mediated regulatory pathways that modulate gene expression and regulate various cellular functions, and are essential for development, differentiation and maturation [61], [62]. The structure of Ago proteins is well conserved, consisting of an amino-terminal domain, the MID domain, and their signature PAZ and Piwi domains [61], [62]. Argonaute proteins form the core of RNA-induced silencing complexes (RISCs). Mice and humans generate four Ago proteins, but only Ago2 appears to retain the ability to cleave RNA targets. Whether the functions of Ago1, Ago3, and Ago4 might differ is not clear [62], [63]. Ago1 and Ago2 are the two proteins that have been more extensively studied. Ago1 selectively binds to microRNAs, while Ago2 binds to both microRNAs and siRNAs [15]. MicroRNA duplexes are intrinsically asymmetric, with the guide strand preferentially entering Ago1-RISC complex, while the miR* strand is usually loaded as functional species into Ago2-RISC complex [15]. It has been shown that the miR* strand can silence its messenger RNA targets both in vitro and in-vivo. Therefore, each microRNA precursor can potentially produce two mature strands (miR and miR*) that are differentially sorted into Ago1-mediated or Ago2-mediated RNA interference (RNAi) pathways, respectively.

In addition to being key components in the RISC complex that are responsible for gene silencing, Ago proteins also play a role in maintaining a proper level of mature miRNA strands, which was initially revealed by gene targeting against Ago1 and Ago2 genes. When Ago1 was depleted, a significant reduction in miR strands and an unexpected concomitant increase in the levels of target messenger RNAs were observed. When Ago2 was depleted, a reduction of endogenous siRNA and miR* levels was observed, however, the miR strand levels were unaffected. Recent studies further indicate that Ago proteins stabilize miRNAs, either within living cells or after the miRNAs are released from cells into the bloodstream [64]–[66].

In the present study, the *in-vitro* transfection experiments suggest that increased expression of Ago1 and/or Ago2 genes contribute to the altered levels of miR-21 and miR-21* in the cells. The in vivo data from mouse hearts revealed age-related changes in the expression of Ago1 and Ago2 genes. However, the Ago1 and miR-21* gene expression were decreased in 24-month-old mouse hearts vs. 4 month-old hearts; while Ago2 expression was increased in 24-month-old mouse hearts vs. 4 month-old hearts. We also observed that some miRNAs were down-regulated in the 24-month-old vs. 4-month-old hearts. It is plausible that the degree of down-regulation of Ago1 and up-regulation of Ago2, the length of the 3′ UTR of the target, the relative abundance of the endogenous miRNA of interest, and the relative abundance of the target messenger RNA might influence the down-regulation of the miRNAs/clusters. In addition, some miRNAs are processed through a non-canonical miRNA biogenesis pathway that might not require Ago proteins [67], [68].

**Figure 16:**
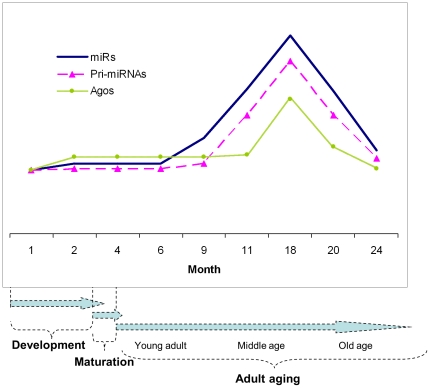
Schematic representation of a model of miRNA expression, which is regulated by transcriptional regulation and other factors, such as Ago proteins. The major change in expression of the miRNA biogenesis components occurred during later adulthood. miRs: both miR and miR*. Pri-miRNAs: the primary miRNA transcripts. Agos: the Argonaute proteins. Month: the mouse age (from 1–24 months in the present study). Adult Aging is a process that spans many decades in human beings and, similarly, many months in rodents. To translate human aging with a much longer life span into rodent years may be fraught with complexities. However, in general, adult aging might be arbitrarily divided into an earlier phase (humans between approximately 20 and 39 years and in C57BL6 mouse strain between 4 and 10 mo), a middle phase (humans between approximately 40 and 59 years and in C57BL6 between approximately 11 and 22 mo), and a later phase (humans between 60 and 79 years and in C57BL6 between 23 and 35 mo). Senescence (the age at which 50% of the populations has died) occurs in humans at around 79–80 yrs, and in C57BL6 mice at around 35–36 months. Of course, with the progressively changing demographics and aging of the population, these arbitrary divisions of what might be considered “early” or “late” aging will likely undergo revision and change.

Taken together, we propose a model of miRNA expression in the aging heart where miRNAs are regulated by the levels of Ago proteins and miRNA primary transcripts. In this model, the Ago proteins bind to the miRNA to stabilize the miRNAs while the pri-miRNA transcripts upregulate synthesis of miRNAs, thereby resulting in a synergistic effect when both are increased (Figure 16).
